# Post Cerebrovascular Stroke Catatonic Psychosis: A Case Report

**DOI:** 10.1192/j.eurpsy.2023.2245

**Published:** 2023-07-19

**Authors:** H. Hasan, M. Abdo, S. Rabei

**Affiliations:** 1 Neuropsychiatry, Helwan University - Faculty of Medicine; 2 Psychological Medicine Hospital, Cairo, Egypt

## Abstract

**Introduction:**

Catatonia due to cerebrovascular stroke is a rare condition that needs further observation and research.

**Objectives:**

To review the opinions of psychotic disorders experts worldwide as to this issue based on evidence and clinical experience and to consider strategies for future investigations.

**Methods:**

This case shows a 64 years old female who suddenly developed wish for isolation, followed 10 days later by discontinuity of ideas, hallucinatory behavior and food refusal. She had verbal and physical aggression due to a fixed belief that family members are conspiring somehow to harm her.

**Results:**

On examination she was mute with waxy flexibility and negativism. Extensor plantar reflex was evident. MRI Brain showed small vessel disease and right basal ganglia acute ischemic infarction. On IV midazolam 7.5 mg, patient’s mutism, negativism and waxy flexibility improved. Lower limb Venous Duplex revealed acute right popliteal and left soleal veins thrombosis. CT angiography showed Bilateral pulmonary embolism with no pulmonary infarction. D dimer was positive.

**Conclusions:**

Early diagnosis and intervention improves outcome if psychiatric teams gives attention and has enough awareness with warning symptoms and prompt necessary interventions.

**Disclosure of Interest:**

None Declared

